# Development and Internal Validation of a New Prognostic Model Powered to Predict 28-Day All-Cause Mortality in ICU COVID-19 Patients—The COVID-SOFA Score

**DOI:** 10.3390/jcm11144160

**Published:** 2022-07-18

**Authors:** Emanuel Moisa, Dan Corneci, Mihai Ionut Negutu, Cristina Raluca Filimon, Andreea Serbu, Mihai Popescu, Silvius Negoita, Ioana Marina Grintescu

**Affiliations:** 1 Department of Anaesthesia and Intensive Care Medicine, Faculty of Medicine, ‘Carol Davila’ University of Medicine and Pharmacy, 020021 Bucharest, Romania; dan.corneci@umfcd.ro (D.C.); mihai.popescu@umfcd.ro (M.P.); silviusneg@gmail.com (S.N.); ioana.grintescu@umfcd.ro (I.M.G.); 2 Clinic of Anaesthesia and Intensive Care Medicine, Elias Emergency University Hospital, 011461 Bucharest, Romania; mnegutu@gmail.com; 3 Clinic of Anaesthesia and Intensive Care Medicine, Dr. Carol Davila Central Military Emergency University Hospital, 010825 Bucharest, Romania; filimon_raluca@yahoo.ro (C.R.F.); andreea.serbu91@gmail.com (A.S.); 4 Clinic of Anaesthesia and Intensive Care Medicine, Fundeni Clinical Institute, 022328 Bucharest, Romania; 5 Clinic of Anaesthesia and Intensive Care Medicine, Clinical Emergency Hospital of Bucharest, 014461 Bucharest, Romania

**Keywords:** COVID-19, SOFA, SARS-CoV-2, ICU, prognostic score, mortality, neutrophil-to-lymphocyte ratio, NLR, ARDS

## Abstract

Background: The sequential organ failure assessment (SOFA) score has poor discriminative ability for death in severely or critically ill patients with Coronavirus disease 2019 (COVID-19) requiring intensive care unit (ICU) admission. Our aim was to create a new score powered to predict 28-day mortality. Methods: Retrospective, observational, bicentric cohort study including 425 patients with COVID-19 pneumonia, acute respiratory failure and SOFA score ≥ 2 requiring ICU admission for ≥72 h. Factors with independent predictive value for 28-day mortality were identified after stepwise Cox proportional hazards (PH) regression. Based on the regression coefficients, an equation was computed representing the COVID-SOFA score. Discriminative ability was tested using receiver operating characteristic (ROC) analysis, concordance statistics and precision-recall curves. This score was internally validated. Results: Median (Q1–Q3) age for the whole sample was 64 [55–72], with 290 (68.2%) of patients being male. The 28-day mortality was 54.58%. After stepwise Cox PH regression, age, neutrophil-to-lymphocyte ratio (NLR) and SOFA score remained in the final model. The following equation was computed: COVID-SOFA score = 10 × [0.037 × Age + 0.347 × ln(NLR) + 0.16 × SOFA]. Harrell’s C-index for the COVID-SOFA score was higher than the SOFA score alone for 28-day mortality (0.697 [95% CI; 0.662–0.731] versus 0.639 [95% CI: 0.605–0.672]). Subsequently, the prediction error rate was improved up to 16.06%. Area under the ROC (AUROC) was significantly higher for the COVID-SOFA score compared with the SOFA score for 28-day mortality: 0.796 [95% CI: 0.755–0.833] versus 0.699 [95% CI: 0.653–0.742, *p* < 0.001]. Better predictive value was observed with repeated measurement at 48 h after ICU admission. Conclusions: The COVID-SOFA score is better than the SOFA score alone for 28-day mortality prediction. Improvement in predictive value seen with measurements at 48 h after ICU admission suggests that the COVID-SOFA score can be used in a repetitive manner. External validation is required to support these results.

## 1. Introduction

The sequential organ failure assessment (SOFA) score is one of the diagnostic criteria for sepsis according to Sepsis-3 [1]. In viral sepsis secondary to severe acute respiratory syndrome coronavirus type 2 (SARS-CoV-2) [2], the SOFA score as a predictor for death or triage for invasive mechanical ventilation need was considered to be inaccurate and, therefore, should be used cautiously [3,4,5,6].

In a cohort of 15,112 patients from 86 United States centers, the preintubation SOFA score had poor discriminant value between survivors and non-survivors. The area under the receiver operating characteristic (AUROC) was inferior to that of age. Age increased the predictive ability when added to the SOFA score, but authors advised caution when the SOFA score is used for mechanical ventilation triage [3].

Raschke et al. mainly emphasized that in critically ill patients with coronavirus disease 2019 (COVID-19), the mainstay of organ dysfunction is respiratory failure, and even critical presentations are mostly considered severe single-organ dysfunction [4]. Contrarily, direct non-respiratory organ involvement was observed for all organ systems introduced in the SOFA score [7,8], but one should question if the parameters or cut-off values introduced in this score correctly evaluate these dysfunctions in critically ill COVID-19 patients [9].

Furthermore, the aforementioned results and observations are conflicting [2,3,4,5,6,7,8]. Henceforth, we consider that a crucial issue is represented by the presence of bacterial co-infection at the time of diagnosis or during ICU stay when using and reporting the SOFA score as a severity or predictive score for COVID-19 in clinical studies.

Thus, partially based on the methodology that led to the development of the chronic liver failure-acute-on-chronic liver failure (CLIF-ACLF) score [10], our aim was to construct a new SOFA-derived score powered to predict 28-day all-cause mortality using the SOFA score and other variables independently associated with death at ICU admission in critically ill COVID-19 patients without bacterial co-infection.

## 2. Materials and Methods

### 2.1. Study Population

Four hundred and twenty-five patients admitted to the intensive care units (ICU) from two tertiary centers (Elias University Emergency Hospital, Bucharest and “Carol Davila” Central Military University Emergency Hospital, Bucharest, Romania), during a period of 19 months (April 2020–November 2021) were included in this retrospective, observational study. The study was approved by the Local Ethics Committees from both centers (2330/2022, 517/2022). Inclusion criteria were age ≥18 years, COVID-19 pneumonia confirmed through real-time polymerase chain reaction (RT-PCR) and chest Rx or CT scan, acute respiratory failure or acute respiratory distress syndrome (ARDS) secondary to COVID-19 pneumonia requiring non-invasive (NIV) or invasive mechanical ventilation (IMV) or high-flow oxygen therapy (HFOT), SOFA score ≥ 2 and an ICU length of stay (ICU-LoS) ≥72 h. Exclusion criteria were patients with confirmed or suspected bacterial co-infection at ICU admission or during the first 48 h after ICU admission (clinical or radiological examination suggestive for bacterial co-infection, positive microbiological cultures), over 48 h between ARDS diagnosis and ICU admission, patients with ongoing chemo-, radio- or immunotherapy, known end-stage organ disease (heart, liver, kidney), patients in whom data could not be collected and patients who were transferred from other ICUs after 48 h from ARDS diagnosis. ARDS was defined according to the Berlin criteria [11]. Patients who developed bacterial or fungal co-infections during ICU stay were not excluded. All patients were treated according to the treatment guidelines published in the Romanian Official Gazette [12]. These protocols were in agreement with the European and international treatment guidelines for COVID-19 patients [12].

### 2.2. Data Collection and Study End-Points 

Data were retrospectively collected at ICU admission and 48 h after ICU admission from patients’ medical files. Demographic (age, gender, body mass index), clinical (associated diseases, vaccination status, level of respiratory support, Glasgow Coma Scale (GCS) score, need for vasoactive agents, bacterial or fungal co-infections acquired during ICU stay, length of stay) and laboratory data were included. The following laboratory parameters were collected: white blood cells, neutrophils, lymphocytes, neutrophil-to-lymphocyte ratio (NLR) and platelet count, creatinine, bilirubin, D-dimers, ferritin, PaO_2_, PaO_2_/FiO_2_ ratio and C-reactive protein (CRP) values. Microbiological screening was performed at ICU admission in order to exclude bacterial co-infection. 

The SOFA score was calculated according to Vincent et al. [13]. The worst values during the first 24 h and at 48 h after ICU admission were used. The level of respiratory support was provided depending on clinical examination, peripheral oxygen saturation (SpO_2_) and blood gas values as decided by the attending physician. Respiratory therapy was applied in a step-wise approach from: (i) HFOT to NIV, (ii) from HFOT or NIV to IMV. Patients underwent invasive mechanical ventilation if one of the following criteria were met: persistent severe hypoxemia (PaO_2_/FiO_2_ < 100) despite maximal respiratory support provided (non-invasive mechanical ventilation (NIV) or HFOT), respiratory support failure (increased work of breathing, fatigue, arterial blood pH < 7.2 due to respiratory acidosis), or neurologic dysfunction secondary to respiratory failure and shock requiring high-dose of vasopressor support. NIV was provided for patients with acute respiratory failure and known respiratory or cardiac conditions (e.g., obstructive pulmonary disease, obstructive sleep apnea, chronic heart failure). Additionally, NIV and HFOT were provided for patients with acute respiratory failure, but that were cooperative and able to maintain their protective reflexes. Lastly, for patients under deep sedation that could not be neurologically evaluated, the Glasgow Coma Scale (GCS) score before endotracheal intubation or before ICU admission was considered. All-cause mortality at 28 days was the primary end-point. For patients discharged from hospital past these end-points, the Romanian Informatics Platform of Health Insurance was used for patient survival status. 

### 2.3. Statistical Analysis

All included parameters were tested for distribution normality using the Kolmogorov–Smirnov test. Categorical variables were expressed as absolute (number) and relative (percentage) frequencies. Continuous variables were expressed as median and interquartile range (IQR: Q1–Q3). Chi-square test was used to compare categorical data after cross-tabs were computed, while for continuous data, Mann–Whitney U (two continuous independent variables), Wilcoxon (two continuous related variables) and Kruskal–Wallis H (more than two continuous independent variables) tests were used. Cox proportional hazards (PH) regression was used to build the predictive models using a stepwise method. Variables were kept in the model if *p* < 0.05 and removed if *p* > 0.1. Variables introduced in the model were: age, NLR, SOFA score, CRP, D-dimers, ferritin and gender. The level of respiratory support was not introduced in the final model, because points were already assigned differently depending on mechanical ventilation need in the SOFA respiratory subscore. Hospital-acquired infections during ICU stay were not introduced as a potential predictor in the final model, because the model was created based on the ICU admission values from patients without bacterial co-infection. The equation for the COVID-SOFA score was computed based on the regression coefficients. The model’s goodness-of-fit was evaluated with the Hosmer–Lemeshow test and the model was considered well-fitted if *p* > 0.05. In order to test which model was better, the Akaike information criterion (AIC), Bayesian information criterion (BIC) and the likelihood ratio (LR) test were used. The model was considered better than the previous if the AIC and BIC were lower and the LR test had a *p* < 0.05. The discriminant ability was assessed with concordance statistics (Harrell’s C-index), receiver operating characteristic (ROC) analysis, precision-recall curves (PRC) and improvement in prediction error rate. Area under the ROC curves (AUROC) was compared between the SOFA score and COVID-SOFA score using the DeLong method [14]. Area under the PRC was compared between the SOFA score and COVID-SOFA score using bias-corrected accelerated bootstrapping (BC(a)) with a resample = 1000 iterations. The difference between the PR curves was considered significant (*p* < 0.05) if both limits of the BC(a)’s 95% confidence interval were >0. Hazard ratio, AUROC, AUPRC, Harrell’s C-index values, regression coefficients and bias were reported together with their 95% confidence intervals (95% CI). Improvement in prediction error rate was calculated using the following formula: 100 × (C-index_COVID-SOFA_ − C-index_SOFA_)/(1 − C-index_SOFA_) and expressed as percentage [10]. Lastly, mortality probability was calculated based on the Cox equation [15]:

S(*t*) = exp (−H_0_(*t*) × PI)

Rewritten for the COVID-SOFA score:

S(*t*) = 1 − e^[−CI(*t*) × exp(^^β^^(*t*) × COVID-SOFA)]^

where *t* = time-point for survival probability (e.g., 28-day); CI(*t*) = baseline cumulative hazard at the given time-point; exp(β(*t*) × COVID-SOFA) = probability index; the expβ(*t*) = the exp(β) for COVID-SOFA score at the given time-point, multiplied by the calculated COVID-SOFA score.

Internal validation was performed by bias-corrected accelerated bootstrapping (resample = 1000 iterations) for calibration, Cox PH regression and PR curves. The model was considered valid if after BC(a), both limits of the 95% BC(a) CI were >0 and the same significant statistical results were maintained. All tests were two-tailed and were considered significant if *p* < 0.05. 

Our findings were reported with respect to the TRIPOD Statement (transparent reporting of a multivariable prediction model for individual prognosis or diagnosis) checklist [16]. The TRIPOD checklist is available in the Supplementary Material (Table S1).

For this study, IBM Statistical Package for Social Sciences (SPSS) for Windows^®^ version 20.0 (Armonk, NY, USA. IBM Corp.) and MedCalc Software^®^, version 20.106 (Ostend, Belgium) were used.

## 3. Results

### 3.1. Baseline Characteristics of the Study Population at ICU Admission

**Figure 1:**
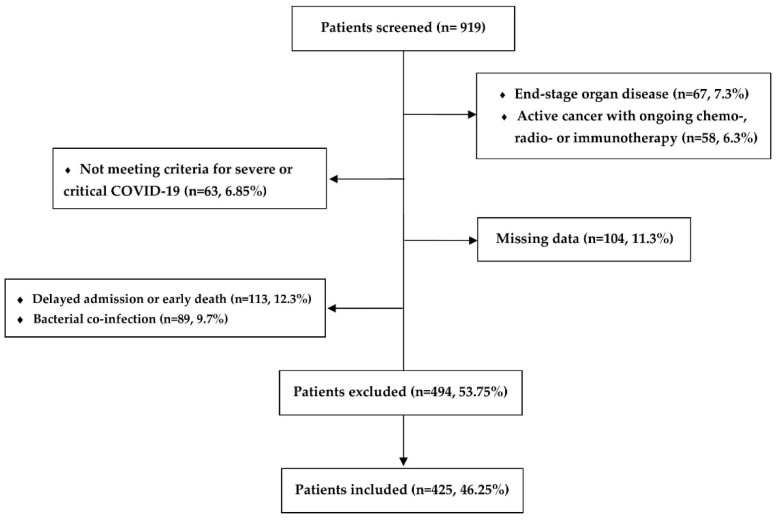
Flowchart of the study population.

For this study, 425 patients met the inclusion criteria (Figure 1). Median age for the whole sample was 64 (55–72), and 68.2% of patients were male. Regarding associated diseases, 297 (69.9%) patients presented obesity, 312 (73.4%) cardiac disease, 155 (36.5%) diabetes mellitus and 52 (12.2%) respiratory disease.

At ICU admission, 51 (12%) patients were under IMV, while 225 (52.9%) and 149 (35.1%) were under NIV and HFOT, respectively. Invasive mechanical ventilation was provided during ICU stay in 211 (56.41%) non-IMV patients. IMV need was significantly higher in NIV patients compared with HFOT patients (*p* < 0.001). Out of the NIV patients at ICU admission, for 158 (70.2%) IMV was provided during ICU stay, while for HFOT patients, IMV was required only in 53 (35.6%) of them. Median SOFA for the whole sample at ICU admission was 4 (3–5) points, with significant differences between patients under IMV, NIV and HFOT (8 (6–9) vs. 4 (3–5) vs. 3 (2–3) points, *p* < 0.001). The median for ICU-LoS was 12 days (8–17). Hospital-acquired infections had a frequency of 208 (48.9%), and the median duration from the time of ICU admission to the time of diagnosis was 7 (6–10) days. For the whole sample, 28- and 60-day all-cause mortality were 54.58% (232) and 57.64% (245), respectively. 

Differences between survivors and non-survivors are presented with respect to 28-day all-cause mortality. Factors associated with increased mortality were age (*p* < 0.001), presence of cardiac disease (*p* < 0.001), diabetes mellitus (*p* < 0.001), pre-existing respiratory disease (*p* = 0.025), higher SOFA scores (*p* < 0.001) and higher levels of respiratory support (87%, 228 out of 262 IMV patients, *p* < 0.001). Moreover, based on SOFA score parameters, non-survivors had significantly lower PaO_2_/FiO_2_ (*p* < 0.001), Glasgow Coma Scale scores (*p* < 0.001) and platelet counts (*p* = 0.01), higher vasopressor support (*p* < 0.001) and higher creatinine values (*p* = 0.01). Furthermore, non-survivors had lower lymphocytes counts (*p* < 0.001) and higher neutrophil counts (*p* = 0.01), neutrophil-to-lymphocyte ratios (*p* < 0.001) and D-dimer values (*p* < 0.001). C-reactive protein and ferritin values at ICU admission were not associated with 28-day all-cause mortality (*p* > 0.05). 

Also, a significant difference was observed regarding ICU-LoS between survivors and non-survivors (*p* = 0.003). Lastly, mortality was higher in patients with hospital-acquired infections (*p* < 0.001). The HAI incidence was 48.9%, Out of patients under invasive mechanical ventilation, 186 (70.7%) had an HAI during ICU stay compared with patients not on IMV (*p* < 0.001). Ventilator-associated pneumonia rate was 60.2% (112 patients) in IMV patients. This group of patients had a higher mortality rate (90 patients, (80%), *p* < 0.001). A summary of the study population characteristics is available in Table 1. 

#### 3.1.1. Building the Predictive Model and the COVID-SOFA Score Equation

Variables were introduced in the predictive models using a stepwise (forward likelihood ratio) method and bias-corrected accelerated bootstrapping. Gender, D-dimers, C-reactive protein and ferritin values were not associated with death in the multivariate analysis. Although hospital-acquired infections during ICU stay independently predicted death, this variable was not introduced in the final model, because the model was created based on the ICU admission values from patients without bacterial co-infection. For the final model, age, NLR and SOFA score were selected by the statistical software. Based on their regression coefficients, the following equation was computed:

COVID-SOFA score = 10 × [0.037 × Age + 0.347 × ln(NLR) + 0.16 × SOFA]

The value obtained in parenthesis is multiplied by 10 and rounded to ease the use of the final score. The full model is available in the Supplementary Materials (Table S2).

#### 3.1.2. COVID-SOFA Score’s Goodness-Of-Fit

**Figure 2:**
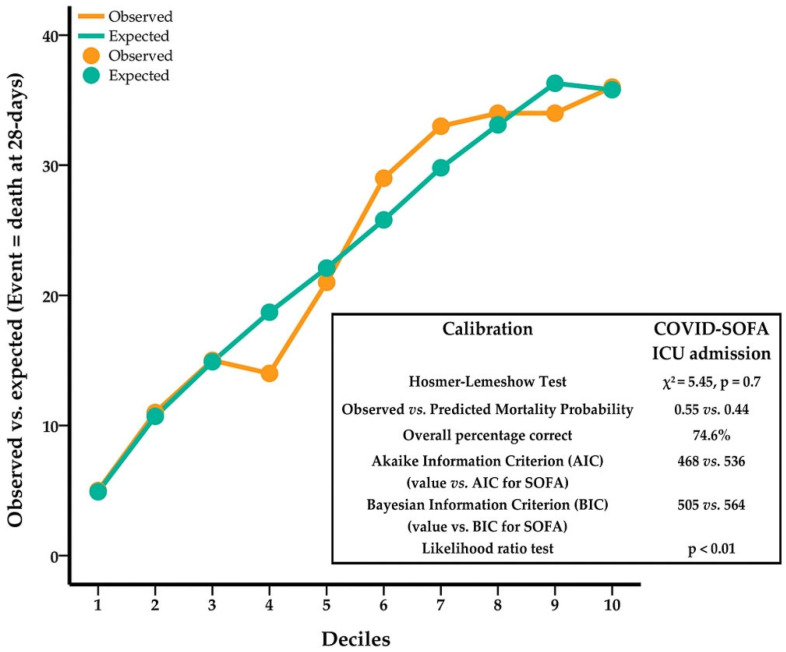
Calibration plot and COVID-SOFA score’s goodness-of-fit.

The final model was well-fitted for 28-day mortality (χ^2^ Hosmer–Lemeshow = 5.45, *p* = 0.7). Observed and predicted probabilities of death did not differ significantly across groups (Figure 2), with an overall percentage of correctly predicted cases of 74.6%. AIC and BIC values were lower for the COVID-SOFA score compared with SOFA score (Figure 2).

### 3.2. COVID-SOFA Score Discriminative Power between Survivors and Non-Survivors at 28 Days

**Figure 3:**
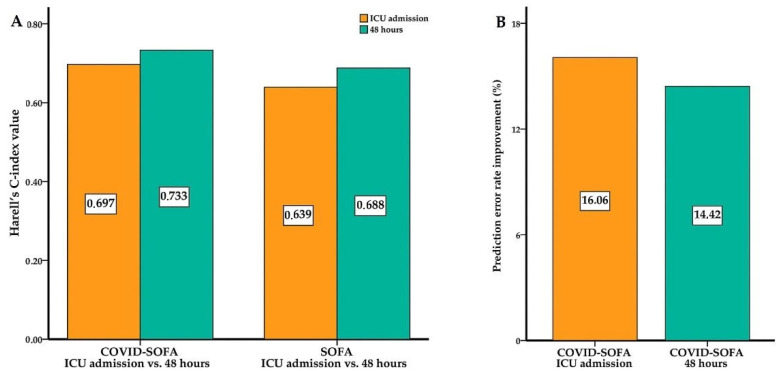
Harrel’s C-index values (**A**) and prediction error rate improvement (**B**). C-index values were higher for COVID-SOFA score compared with SOFA score alone at ICU admission and 48 h (**A**), and this was equivalent to an improvement in the prediction error rate for 28-day all-cause mortality (**B**).

Harrell’s C-index value for 28-day all-cause mortality was 0.697 (95% CI; 0.662–0.731) for the COVID-SOFA score at ICU admission and 0.639 (95% CI: 0.605–0.672) for the SOFA score at ICU admission, respectively. Thus, an increase of 0.058 in C-index value was observed with this new model. This was equivalent to an improvement in the prediction error rate of 16.06% (Figure 3A).

Lastly, ROC analysis and precision-recall curves were computed to test the discriminative power of the COVID-SOFA score compared with the SOFA score alone. The observed AUROC values for 28-day all-cause mortality for the COVID-SOFA score versus SOFA score at ICU admission were 0.796 (95% CI: 0.755–0.833) versus 0.699 (95% CI: 0.653–0.742), *p* < 0.001. The AUPRC for COVID-SOFA score was 0.813 (95% CI: 0.757–0.858) with a significant difference between the AUPRC of the two scores of 0.079 (95% BC(a) CI: 0.066–0.094) (Table 2).

**Figure 4:**
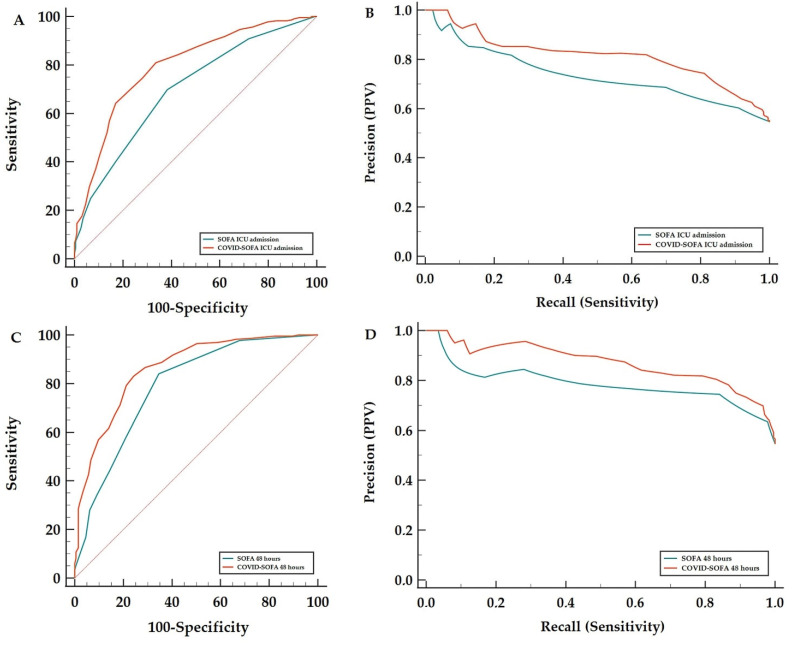
Area under the receiver operating characteristic (AUROC) (**A**,**C**) and precision-recall curves (AUPRC) (**B**,**D**) for SOFA and COVID-SOFA scores at ICU admission and 48 h regarding 28-day all-cause mortality (**C**,**D**). AUROC and AUPRC were significantly higher for the COVID-SOFA score compared with the SOFA score alone.

Higher Harrell’s C-index, AUROC and AUPRC values were observed for repeated measurements at 48 h after ICU admission, as described in Table 2 and Figure 3 and Figure 4. A C-index value of 0.733 (95% CI: 0.700–0.765) was obtained for the COVID-SOFA score at 48 h which was 0.045 higher than the C-index value obtained for the SOFA score at 48 h (Figure 3). The AUROC values for the COVID-SOFA score versus SOFA score at 48 h were: 0.862 (95% CI: 0.826–0.893) versus 0.788 (95% CI: 0.746–0.826), *p* < 0.001. Regarding the AUPRC at 48 h, the difference between the two scores was significant: 0.086 (95% BC(a) CI: 0.07–0.11) (Table 2, Figure 4). 

A brief analysis of the 60-day all-cause mortality based on the 28-day all-cause mortality model is available in Supplementary Material (Table S3). 

### 3.3. Mortality Probability Calculation at All End-Points Using the COVID-SOFA Score

The mortality probability at the studied end-points can be calculated using the Cox equation rewritten for the COVID-SOFA score [15]: S(*t*) = 1 − e^[−CI(*t*) × exp(β(*t*) × COVID-SOFA)]^

The coefficients for COVID-SOFA score at ICU admission:

CI(28-day) = 0.017; exp(β)(28-day) = 1.1045

The coefficients for COVID-SOFA score at 48 h:

CI(28-day) = 0.010; exp(β)(28-day) = 1.1134

## 4. Discussion

Our study describes the development and internal validation of a new predictive model in 425 critically ill patients with COVID-19. A score that has accurate predictive value in COVID-19 critically ill patients is necessary given the high mortality rates even in patients considered to receive the best-of-care. 

In our cohort, demographic characteristics were similar to those of other study cohorts, non-survivors being older and having more comorbidities [17,18]. Regarding hematological changes induced by SARS-CoV-2, these were similar with previously reported data, with lymphopenia, neutrophilia and higher D-dimer values being associated with poor outcome [19,20]. The SOFA score values in COVID-19 patients admitted to ICU have important variations between studies [19], depending on: (i) the selected patients, (ii) the moment of measurement (e.g., ICU admission, before endotracheal intubation, repeated measurements), and (iii) missing data and how points were assigned [21,22,23,24,25]. In our study, the median SOFA score at ICU admission was significantly different depending on the level of respiratory support required. This observation is supported by results from other published data [9]. The median SOFA score for IMV patients in our study was 8 (6–9) points, compared with median SOFA scores of 6 to 10 points reported by other authors [26,27,28]. Moreover, increased heterogeneity was observed in the reported SOFA score values between survivors and non-survivors [26,27,28,29]. For patients requiring NIV or HFOT, the median SOFA scores were 4 (3–5) and 3 (2–3) points, and this was similar for other study populations in which the mean SOFA score values ranged between 2.4 and 4.8 [22,29] points. Lastly, the presence of bacterial co-infection can modify the SOFA score values, as bacterial and viral sepsis have different features [2] and the SOFA score was mainly studied in bacterial sepsis. 

As stated, we partially built this model based on the methodology of the CLIF-ACLF score [10]. In contrast to the CLIF-SOFA score, parameters for organ failure were not changed and organ failure was not redefined. In order to build the predictive model, after stepwise multivariate Cox PH regression was performed, the SOFA score independently predicted death together with age and NLR. Both age and NLR are considered to have independent predictive value for death [17,18,19], progression to severe or critical disease [19,30] and need for invasive mechanical ventilation [19] in COVID-19 patients. Moreover, given the fact that age and NLR have independent prognostic value in bacterial sepsis [31,32], we can speculate that this new score is reproducible in patients with COVID-19 and bacterial co-infection.

From a pathophysiological perspective, NLR was used instead of lymphocytes and/or neutrophils alone. Lymphocytes and neutrophils play a pivotal role in viral sepsis regarding systemic inflammation, immunothrombosis and disease progression [33,34,35]. Based on the published data [20,33,34,35], quantitative and qualitative alterations of lymphocytes can be considered as a separate organ dysfunction in viral sepsis secondary to SARS-CoV-2 infection. Lastly, NLR values are highly correlated with C-reactive protein and D-dimer values [19]. 

Results from concordance statistics showed that the COVID-SOFA score predictive value was superior to that of the SOFA score alone (Harrell’s C-index increased between 0.045–0.058) and improved the prediction error rate (between 14.42–16.06%). Furthermore, the discriminative ability of the COVID-SOFA score (evaluated with ROC analysis) increased the AUROC significantly. The increases in AUROC for the new model were between 0.074 and 0.097. The same observations were shown for AUPRC, with significant differences between the COVID-SOFA score and SOFA score at ICU admission and 48 h. 

In our study, AUROC for SOFA at ICU admission was similar to that reported by Keller et al. [3] (0.69 (95% CI: 0.66–0.73) versus 0.66 (95% CI: 0.65–0.67)). The authors suggested that the AUROC increased to 0.74 when age was added [3]. Poor discriminant value for mortality or triage for mechanical ventilation in critically ill COVID-19 patients was reported by other authors, too [4]. These data led to ethical concerns when using the SOFA score to decide to whom a ventilator would be allocated in a crisis management protocol [5,6]. Tolchin et al. observed that in triage protocols that use the SOFA score, Non-Hispanic Black patients were more likely to have higher SOFA scores and restricted access to ICU beds or ventilators [22]. The goal should be to create a better predictive model with an improved prediction error rate that will further reduce biased decisions regarding the care provided to critically ill COVID-19 patients [5].

Moreover, repeated measurements at 48 h after ICU admission showed an even better predictive ability of the COVID-SOFA score with an increase in the C-index of up to 0.058 and an improvement in prediction error rate of up to 16.06% compared with the SOFA score alone for 28-day all-cause mortality. Discriminant ability was very good for the COVID-SOFA score at 48 h with an increase in AUROC of 0.074 and in AUPRC of 0.086. This suggests that the COVID-SOFA score is better to be used as a repetitive score, as recommended with the original SOFA score [36]. Lastly, dynamic changes in NLR [19] and the SOFA score [23,24] are associated with disease severity and death. 

Several prognostic scores for mortality prediction in COVID-19 patients admitted to ICU have been proposed [37,38,39,40,41]. Some of them were built using machine learning, which led to the development of complex models using large amounts of data [37,38,39,40]. These scores included demographic, clinical, radiological and laboratory variables and had better discriminative ability compared to other prognosis or severity scores used in critically ill COVID-19 patients. The intubated COVID-19 predictive (ICOP) score was designed for early mortality prediction in intubated COVID-19 patients. Following concordance statistics and ROC analysis, the score’s discriminative power for 14-day mortality was superior to that of the SOFA and CURB-65 scores: ICOP AUROC = 0.70 (95% CI: 0.66–0.75) versus SOFA AUROC = 0.65 (95% CI: 0.61–0.70) [41]. The Survival of Severely Ill COVID (SOSIC) scores were build to predict 90-day mortality in patients with COVID-19 after 1 to 2 weeks in ICU [40]. The SOSIC-1 score takes into account, among other parameters, the respiratory, cardiovascular and renal subscores of the SOFA score and the lymphocyte count at ICU admission. Furthermore, their study population included patients with the same respiratory support levels as in our cohort. The discriminative ability for 90-day mortality was good with an AUROC of 0.76 (95% CI: 0.711–0.808). Moreover, in their model, co-infection at ICU admission was introduced as an independent variable [40]. 

External validation of 32 scores designed to predict in-hospital mortality, ICU admission or invasive mechanical ventilation need was performed on a cohort of 14,343 patients [42]. Out of 32 scores, 19 had a significantly lower predictive ability compared with the original reports, seven had an AUROC > 0.75 to discriminate in-hospital mortality, while for the composite outcome (in-hospital mortality or ICU admission), only two scores had an AUROC ≥ 0.7: CORONATION-TR [43] (AUROC = 0.724 (95% CI: 0.714–0.733) and COVID-GRAM [44] (AUROC = 0.700 (95% CI: 0.690–0.711)). qSOFA and SIRS scores had very poor predictive value for in-hospital mortality, with AUROC values < 0.6. Furthermore, the authors observed that age was the most important predictive factor in the scores with the highest discriminative ability [42]. Compared with the data reported in the present manuscript, our score performed similarly to or better than the aforementioned scores, but external validation is mandatory to support our results. 

Regardless of our results, one should question if the current modality of evaluating organ dysfunction within the SOFA score is adequate in critically ill COVID-19 patients and if it is limited only to the SOFA score’s six organ systems [9]. For example, neurological dysfunction in COVID-19 patients is strongly associated with death, but its clinical picture is heterogenous, and the GCS score may not be the best tool to assess this dysfunction [45]. Moreover, moderate to severe anxiety at ICU admission is associated with the development of new organ failure in critically ill patients, regardless of critical illness severity [46]. Furthermore, mechanically ventilated COVID-19 patients require unusually high doses of sedatives and opioids [47]. Their use is negatively correlated with systolic and diastolic blood pressure in critically ill patients [48] and is associated with vasopressor agent use [49]. This is also a matter of concern regarding cardiovascular failure assessment in these patients. 

The strengths of our study are represented by: (i) the potential for reproducibility of this score given that patients with different respiratory support levels were included, (ii) selecting patients only with COVID-19 viral sepsis makes this new score a potential tool in prognosis evaluation of viral sepsis in other viral etiologies, (iii) the score includes simple and readily available parameters that are used in a repetitive manner in critically ill patients. The significant increase in mortality prediction with 48 h repeated measurement makes this score promising for serial evaluation of critically ill COVID-19 patients. 

Our study has some limitations. Firstly, the study design was not ideal because of the retrospective nature, lack of racial diversity, and patients were from only two tertiary centers. This increases the risk of bias, and assumptions for different populations can not be made. Secondly, our cohort included patients without confirmed or suspected bacterial co-infection at ICU admission or the first 48 h after ICU admission, but the frequency of bacterial co-infection developed during ICU stay was high. This aspect is important, as HAIs have independent predictive value for death in critically ill COVID-19 patients [50]. We did not study the COVID-SOFA score’s discriminant ability by repeated measurements at the moment of bacterial co-infection diagnosis. Healthcare-associated infections are identified in up to 50% of critically ill COVID-19 patients [50,51,52], and the mortality rate is doubled by their presence [50]. Higher respiratory support need, broad-spectrum antibiotic use at ICU admission, low ICU healthcare professional/patient ratio, low adherence to HAI preventive measures by non-ICU personnel working in COVID ICUs, and delayed ICU admission due to limited ICU bed surge capacity were identified to be associated with increased HAI rates [50,51,52,53]. Among the factors that could explain the high HAI rate in our cohort and subsequently, the high mortality rate in IMV patients, (i) patients included were from the main pandemic peak waves, and ICU bed availability was reduced compared with the number of patients requiring ICU admission; (ii) ICU healthcare professional/patient ratios were low in the most severe peak waves; (iii) the pressure for antibiotic use was high; and (iv) most of the patients required higher respiratory support. Given the fact that an important percentage of patients develop hospital-acquired infections, there is a need to study the predictive value of this new score in these patients as well. Another limitation is represented by the reduced number of vaccinated patients and the subsequent inability to make assumptions about this population. Moreover, cytokines (e.g., interleukin-6) were not routinely measured, thus their predictive value could not be studied in our cohort. Lastly, even if the results are promising, this new score needs external validation on a different cohort. Given the aforementioned limitations and that the outcome studied was 28-day all-cause mortality in a specific population of COVID-19 patients, our results should be interpreted with caution and used as a complementary tool for risk stratification at the time of ICU admission.

## 5. Conclusions

We developed a new prognostic score powered to predict 28-day all-cause mortality in patients with COVID-19 admitted in ICUs from two tertiary centers. The following independent predictors at ICU admission were identified in our study population: age, NLR and SOFA score. The parameters needed to calculate this score are readily available, and they take into account demographic, clinical and laboratory data. Compared with the SOFA score alone, the COVID-SOFA score increased significantly the discriminant ability between survivors and non-survivors at 28 days and subsequently, the prediction error rate was better. If externally validated, this score can represent a complementary tool that can further help clinicians improve risk stratification and decision making regarding the therapeutical interventions needed in COVID-19 patients admitted to ICUs. One should use our score with caution given that it was based on ICU admission parameters, it predicts all-cause mortality, and no external validation was performed.

## Figures and Tables

**Table 1:** Baseline characteristics of the study population at ICU admission.

*n* (%) or Median (IQR: Q1–Q3)	Total Sample	Survivors	Non-Survivors	*p* Value
Demographic and Associated Diseases Data				
Age	64 [55–72]	57 [49.5–65.5]	68 [62–75]	<0.001 *
Gender (Male)	290 (68.2%)	135 (46.6%)	155 (53.4%)	0.489 **
Obesity	297 (69.9%)	127 (42.8%)	170 (57.2%)	0.095 **
Cardiac disease	312 (73.4%)	120 (38.5%)	192 (61.5%)	<0.001 **
Respiratory disease	52 (12.2%)	13 (25%)	39 (75%)	0.025
Diabetes mellitus	155 (36.5%)	53 (34.2%)	102 (65.8%)	<0.001 **
CKD	39 (9.2%)	15 (38.5%)	24 (61.5%)	0.360 **
Vaccinated	26 (6.12%)	11 (42.3%)	15 (57.7%)	0.743 **
SOFA Score Parameters				
PaO_2_/FiO_2_	110 [87–154.5]	124 [95–175]	102.5 [78–140]	<0.001*
IMV	51 (12%)	6 (11.8%)	45 (88.2%)	<0.001 **
NIV	225 (52.9%)	86 (38.2%)	139 (61.8%)	
HFOT	149 (35.1%)	101 (67.8%)	48 (32.2%)	
Creatinine (mg/dL)	0.8 [0.7–1]	0.78 [0.68–0.97]	0.85 [0.7–1.1]	0.01 *
Bilirubin (mg/dL)	0.6 [0.41–0.86]	0.6 [0.41–0.8]	0.6 [0.43–0.87]	0.793 *
Glasgow Coma Scale score	15 [15–15]	15 [15–15]	15 [13–15]	<0.001 *
Noradrenaline use	59 (13.9%)	14 (23.7%)	45 (76.3%)	<0.001 **
Platelet count (×10^3^/μL)	250 [188.5–316.5]	263 [197.5–331.5]	236.5 [170–297]	0.01 *
SOFA	4 [3–5]	3 [2–4]	4 [3–5.75]	<0.001 *
SOFA IMV patients	8 [6–9]	7.5 [7–8]	8 [6–9]	<0.001 ***
SOFA NIV patients	4 [3–5]	4 [3–5]	4 [4–5]	
SOFA HFOT patients	3 [2–3]	2 [2–3]	3 [2–3]	
Respiratory Outcome				
Progression to IMV a	211 (56.41%)	28 (13.28%)	183 (86.72%)	<0.001 **
HFOT to IMV b	53 (35.6%)	6 (11.33%)	47 (88.67%)	<0.001 **
NIV TO IMV c	158 (70.2%)	22 (13.93%)	136 (86.07%)	<0.001 **
Hematological and Inflammatory Parameters				
Lymphocyte count (×10^3^/μL)	0.74 [0.53–1.01]	0.88 [0.6–1.2]	0.67 [0.47–0.87]	<0.001 *
Neutrophil count (×10^3^/μL)	8.53 [6.13–11.65]	8.04 [5.9–11.06]	8.91 [6.47–12.58]	0.01 *
NLR	11.2 [7.21–18.69]	9.19 [5.9–11.06]	14.19 [8.98–20.4]	<0.001 *
WBC count (×10^3^/μL)	9.74 [7.3–12.92]	9.71 [7.16–12.47]	9.81 [7.42–13.74]	0.11 *
D-dimers (ng/mL)	431 [263.3–887.5]	363 [207–684.5]	511 [320.75–987]	<0.001
C-reactive protein (mg/L)	121.7 [64.7–206]	114.5 [63–196.75]	129.9 [67.5–213.2]	0.177
Ferritin (ng/mL)	1018 [572–1483]	941 [494–1460]	1063 [606–1500]	0.229
ICU LoS	12 [8–17]	13 [9.5–17]	11 [7–16]	0.003 *
HAIs	208 (48.9%)	54 (26%)	154 (74%)	<0.001 **
Mortality				
28-day all-cause mortality	232 (54.58%)			
60-day all-cause mortality	245 (57.64%)			

*^a^* (% out of non-IMV patients), *^b^* (% out of HFOT patients), *^c^* (% out of NIV patients), * Mann–Whitney U test, ** Chi-square test; *** Kruskal–Wallis test; IQR = interquartile range, Q1–Q3 = quartile 1 and 3, CKD = chronic kidney disease, PaO_2_ = arterial oxygen partial pressure, FiO_2_ = inspired oxygen fraction, SpO_2_ = peripheral oxygen saturation, IMV = invasive mechanical ventilation, NIV = non-invasive mechanical ventilation, HFOT = high-flow oxygen therapy, SOFA = Sequential Organ Failure Assessment, NLR = neutrophil-to-lymphocyte ratio, WBC = white blood cells, ICU-LoS = intensive care unit length of stay, HAIs = hospital-acquired infections.

**Table 2:** A comparative analysis of COVID-SOFA score vs. SOFA score discriminative ability.

28-Day All-Cause Mortality								
	C-Index, 95% CI	C-Index Diff.	Error Rate Improvement	AUROC 95% CI	AUROC Diff.	*p*	AUPRC 95% CI	AURPC Diff. 95% BC(a) CI
COVID-SOFA ICU Admission	0.697 0.662–0.731	0.058	16.06%	0.796 0.755–0.833	0.097	<0.001 *	0.813 0.757–0.858	0.079
SOFA ICU Admission	0.639 0.605–0.672			0.699 0.653–0.742			0.734 0.674–0.787	0.066–0.094
COVID-SOFA 48 h	0.733 0.700–0.765	0.045	14.42%	0.862 0.826–0.893	0.074	<0.001 *	0.870 0.820–0.907	0.086
SOFA 48 h	0.688 0.654–0.723			0.788 0.746–0.826			0.784 0.727–0.832	0.07–0.11

* DeLong (14); COVID-SOFA = coronavirus disease 2019—sequential organ failure assessment, ICU = intensive care unit, diff. = difference, AUROC = area under the receiver operating characteristic curve, AUPRC = area under the precision-recall curve, 95% CI = 95% confidence interval, BC(a) = bias-corrected accelerated bootstrapping.

## Data Availability

The data presented in this study are available on reasonable request from the corresponding author.

## Supplementary Materials

The following supporting information can be downloaded at: https://www.mdpi.com/article/10.3390/jcm11144160/s1, Table S1: TRIPOD Checklist; Table S2: The full Cox proportional hazards regression model for 28-day mortality; Table S3: Utilization of the 28-day mortality model for 60-day mortality prediction.
