# Supplementary material for: Development and Internal Validation of a New Prognostic Model Powered to Predict 28-Day All-Cause Mortality in ICU COVID-19 Patients—The COVID-SOFA Score
Source: J Clin Med. 2022 Jul 18;11(14):4160. doi: 10.3390/jcm11144160 (PMC9323813; doi:10.3390/jcm11144160)
Supplement: Supplementary file 1 [file jcm-11-04160-s001.zip › Supplementary material S2.pdf]

Table S2a. Final Cox PH regression with bias corrected accelerated bootstrapping

Event = death at 28-days after ICU admission	Stepwise Cox PH regression				Bias corrected accelerated (BC(a)) bootstrap (resample = 1000 cases)			
**Covariate**	**β**	**p**	**HR**	**95% CI of Exp(b)**	**β**	**Bias**	**p**	**95% CI for BC(a)**
**Age**	0.037	< 0.001	1.038	1.026 – 1.05	0.037	0.001	0.001	0.025 – 0.052
**ln(NLR)**	0.347	0.001	1.415	1.167 – 1.717	0.347	0.002	0.002	0.181 – 0.597
**SOFA ICU admission**	0.16	< 0.001	1.176	1.107 – 1.248	0.16	0.001	0.001	0.095 – 0.214

The event was coded with 1 and represented death in the time range between ICU admission and 28-days after ICU admission; PH = proportional hazards, BC(a) = bias corrected accelerated; β = regression coefficient; HR = hazard ratio; 95% CI = 95% confidence interval; ln = natural logarithm; NLR = neutrophil-to-lymphocyte ratio; SOFA = Sequential Organ Failure Assessment; ICU = intensive care unit

Table S2b Variables removed from the Cox PH regression model

	Variables not in the Equation			Model if Term Removed	
		**Score**	**p**	**Term Removed**	**p**
**Step 1**	**ln(NLR)**	**22.585**	**< 0.001**	**Age**	**< 0.001**
	**SOFA ICU admission**	**39.029**	**< 0.001**		
	C-reactive protein	1.062	0.303		
	D-dimers	0.434	0.510		
	Ferritin	1.382	0.240		
	Gender	0.147	0.701		
**Step 2**	**ln(NLR)**	**11.814**	**0.001**	**Age**	**< 0.001**
	C-reactive protein	0.563	0.453	**SOFA ICU admission**	**< 0.001**
	D-dimers	0.229	0.632		
	Ferritin	0.261	0.609		
	Gender	0.045	0.831		
**Step 3**	C-reactive protein	0.016	0.900	**Age**	**< 0.001**
	D-dimers	0.113	0.737	**ln(NLR)**	**0.001**
	Ferritin	0.121	0.728	**SOFA ICU admission**	**< 0.001**
	Gender	0.575	0.448		

Table S2c. Univariate Cox PH regression

Event = death at 28-days after ICU admission	Univariate Cox PH regression			
**Covariate**	**β**	**p**	**HR**	**95% CI of Exp(b)**
**Age**	0.043	< 0.001	1.044	1.033 – 1.056
**ln(NLR)**	0.593	0.001	1.809	1.504 – 2.176
**SOFA ICU admission**	0.219	< 0.001	1.244	1.176 – 1.317

The event was coded with 1 and represented death in the time range between ICU admission and 28-days after ICU admission; PH = proportional hazards, β = regression coefficient; HR = hazard ratio; 95% CI = 95% confidence interval; ln = natural logarithm; NLR = neutrophil-to-lymphocyte ratio; SOFA = Sequential Organ Failure Assessment; ICU = intensive care unit
