# Supplementary material for: Development and Internal Validation of a New Prognostic Model Powered to Predict 28-Day All-Cause Mortality in ICU COVID-19 Patients—The COVID-SOFA Score
Source: J Clin Med. 2022 Jul 18;11(14):4160. doi: 10.3390/jcm11144160 (PMC9323813; doi:10.3390/jcm11144160)
Supplement: Supplementary file 1 [file jcm-11-04160-s001.zip › Supplementary material S3.pdf]

Table S3a. COVID-SOFA score's goodness-of-fit for 60-day mortality based on the 28-day mortality model

60-day mortality	
	**COVID-SOFA ICU admission**
Hosmer-Lemeshow Test	$\chi^2 = 11.45$, p = 0.17
Observed *vs.* Predicted Mortality Probability	0.58 *vs.* 0.46
Overall percentage correct	75.5%
Akaike Information Criterion (AIC) (value *vs.* AIC for SOFA)	455 *vs.* 528
Bayesian Information Criterion (BIC) (value vs. BIC for SOFA)	492 *vs.* 556
Likelihood ratio test	p< 0.01

COVID-SOFA = coronavirus disease 2019 – sequential organ failure assesment, ICU = intensive care unit

Table S3b. A comparative analysis of COVID-SOFA score *vs.* SOFA score discriminative ability for 60-day mortality based on the 28-day mortality model

60-day mortality								
	**C-index 95% CI**	**C-index diff.**	**Error rate improvement**	**AUROC 95% CI**	**AUROC diff.**	**p**	**AUPRC 95% CI**	**AURPC diff. 95% BC(a) CI**
**COVID-SOFA ICU admission**	0.707<br>0.674-0.739	0.061	17.23%	0.802<br>0.761-0.839	0.102	< 0.001*	0.831<br>0.779-0.873	0.076<br>0.063-0.09
**SOFA ICU admission**	0.646<br>0.614-0.678			0.699<br>0.653-0.743			0.755<br>0.697-0.805	
**COVID-SOFA 48 hours**	0.747<br>0.716-0.778	0.049	16.22%	0.872<br>0.837-0.902	0.079	< 0.001*	0.888<br>0.842-0.922	0.084<br>0.068-0.103
**SOFA 48 hours**	0.698<br>0.665-0.732			0.793<br>0.751-0.831			0.803<br>0.749-0.848	

*DeLong (12); COVID-SOFA = coronavirus disease 2019 – sequential organ failure assessment, ICU = intensive care unit, diff. = difference, AUROC = area under the receiver operating characteristic curve, AUPRC = area under the precision-recall curve, 95% CI = 95% confidence interval, BC(a) = bias corrected accelerated bootstrapping
